# Role of interferon-gamma release assays in the diagnosis of pulmonary tuberculosis in patients with advanced HIV infection

**DOI:** 10.1186/1471-2334-10-75

**Published:** 2010-03-20

**Authors:** Adithya Cattamanchi, Isaac Ssewenyana, J Lucian Davis, Laurence Huang, William Worodria, Saskia den Boon, Samuel Yoo, Alfred Andama, Philip C Hopewell, Huyen Cao

**Affiliations:** 1 Division of Pulmonary and Critical Care Medicine, University of California, San Francisco, San Francisco, USA; 2 Francis J. Curry National Tuberculosis Center, University of California, San Francisco, San Francisco, USA; 3 Joint Clinical Research Centre, Kampala, Uganda; 4 HIV/AIDS Division, University of California, San Francisco, San Francisco, USA; 5 California Department of Public Health, Richmond, USA; 6 Faculty of Medicine, Makerere University, Kampala, Uganda; 7 Makerere University-University of California San Francisco Research Collaboration, Kampala, Uganda

## Abstract

**Background:**

T-cell interferon-gamma release assays (IGRAs) may have a role in the diagnosis of active tuberculosis when evaluating patients for whom standard microbiology has limited sensitivity. Our objective was to examine the accuracy of a commercial IGRA for diagnosis of active tuberculosis in HIV-infected persons.

**Methods:**

We enrolled HIV-infected patients admitted to Mulago Hospital in Kampala, Uganda with cough ≥ 2 weeks. All patients underwent standard medical evaluation. We collected peripheral blood specimens at enrollment and performed a commercial, ELISPOT-based IGRA according to the manufacturer's recommendations. IGRA sensitivity and specificity were determined using mycobacterial culture results as the reference standard.

**Results:**

Overall, 236 patients were enrolled. The median CD4+ T-lymphocyte count was 49 cells/μl and 126 (53%) patients were diagnosed with active pulmonary tuberculosis. IGRAs were not performed in 24 (10%) patients due to insufficient mononuclear cell counts. In the remaining 212 patients, results were indeterminate in 54 (25%). IGRAs were positive in 95 of 158 (60%) patients with interpretable results. The proportion of positive test results was similar across CD4+ count strata. IGRA sensitivity was 73% and specificity 54%. IGRA results did not meaningfully alter the probability of active tuberculosis in patients with negative sputum smears.

**Conclusions:**

An ELISPOT-based IGRA detected a high prevalence of latent tuberculosis infection in a hospitalized population of tuberculosis suspects with advanced HIV/AIDS but had limited utility for diagnosis of active tuberculosis in a high prevalence setting. Further research is needed to identify stronger and more specific immune responses in patients with active tuberculosis.

## Background

T-cell interferon-gamma release assays (IGRAs) measure interferon-gamma release by sensitized T-lymphocytes stimulated with *Mycobacterium tuberculosis *(*M. TB*)-specific antigens. Though IGRAs are highly accurate for diagnosis of latent tuberculosis infection (LTBI) [1], their use as a diagnostic tool for active tuberculosis (TB) poses several challenges. IGRAs measure the host immune response to *M. TB *rather than the presence or absence of the organism in clinical specimens. In addition, IGRAs cannot distinguish an immune response to current active TB from an immune response to prior disease or latent infection [2]. However, standard microbiologic tests (smear microscopy, nucleic acid amplification tests, and mycobacterial culture) also have well known limitations, particularly in patients co-infected with HIV [3,4]. Such patients more commonly present with atypical clinical and radiographic findings and pauci-bacillary disease [5]. The consequences of missing a diagnosis are also greater, as the disease is more likely to progress rapidly [6].

We hypothesized that the high sensitivity of IGRAs for detecting *M. TB *infection may help clinicians rule out a diagnosis of active TB in patients co-infected with HIV. In previous studies of HIV-infected adults, the sensitivity of commercial IGRAs for diagnosing active TB has ranged from 85-93%. [7-10] However, none of these studies were conducted in high TB prevalence settings or in patients with advanced HIV-related immunosuppression. In addition, the clinical utility of IGRAs in smear-negative TB suspects has not been assessed adequately. In smear-negative patients, a negative IGRA result might decrease the probability of TB sufficiently to allow clinicians to withhold empiric TB therapy and/or pursue alternative diagnoses.

To address our hypothesis, we conducted a prospective, blinded evaluation of T-SPOT*.TB*^® ^(Oxford Immunotec, Oxford, UK), an FDA-approved, enzyme-linked immunospot (ELISPOT)-based IGRA, for the diagnosis of pulmonary TB in HIV-infected TB suspects admitted to Mulago Hospital in Kampala, Uganda. We chose to evaluate an ELISPOT-based IGRA due to higher sensitivity compared with enzyme-linked immunosorbent assay (ELISA)-based tests. [11-13]

## Methods

### Study population

We screened consecutive patients admitted to the medical wards of Mulago Hospital in Kampala, Uganda to identify those persons presenting with cough ≥ 2 weeks duration (defined as pulmonary TB suspects). We enrolled all pulmonary TB suspects who were HIV-infected, not on anti-TB treatment, and provided informed consent. We excluded patients from this analysis if sputum acid-fast bacillus (AFB) smear results were unavailable or TB status could not be established due to mycobacterial culture contamination (at least two negative cultures were required to exclude TB). Institutional review boards at Makerere University, Mulago Hospital, the Uganda National Council for Science and Technology, and the University of California, San Francisco approved the study protocol.

### Patient evaluation

All patients underwent standard medical evaluation. We collected sputum specimens at enrollment (on the morning after hospital admission) and on the subsequent morning for AFB smear examination (direct Ziehl-Neelsen microscopy) and Lowenstein-Jensen culture, as previously described [14]. All patients with negative AFB microscopy results underwent bronchoscopy with bronchoalveolar lavage (BAL) if referred by the treating ward physician. Trained laboratory technicians examined BAL samples for the presence of mycobacteria (AFB smear examination and Lowenstein-Jensen culture), *Pneumocystis jirovecii*, and other fungi [15]. We determined CD4+ T-lymphocyte counts in all enrolled patients.

### T-SPOT.*TB *assays

We performed and interpreted all assays according to the manufacturer's recommendations. At the time of enrollment, a study officer collected approximately 16 mL of blood for the T-SPOT.*TB *assay in anticoagulant-citrate-dextrose tubes. Trained laboratory technicians at the Joint Clinical Research Centre (JCRC) who were blinded to patients' clinical status processed all blood samples within 6 hours of collection. Peripheral blood mononuclear cells (PBMCs) were isolated using ficoll-hypaque gradient centrifugation and cell count and viability were determined using a Guava automated counter (Guava Technologies, Hayward, CA). IGRAs were performed only when PBMC viability was greater than 80%. Spots were counted using an automated ELISPOT reader (Immunospot Analyzer, Cellular Technologies, Ltd. Cleveland, OH). We considered IGRA results to be positive if: 1) the negative control had < 5 spots and wells stimulated with test Panel A (containing ESAT-6 antigen) or Panel B (containing CFP-10 antigen) had 6 or more spots or 2) the negative control had 5-10 spots and wells stimulated with test Panel A or Panel B had at least twice the number of spots in the negative control. We considered IGRA results to be indeterminate if the negative control spot count was > 10 (high background) or if the positive mitogen control spot count was < 20.

### Outcome classification

We defined the reference standard for active TB as a positive mycobacterial culture result on any sputum or BAL fluid specimen (culture-positive TB). Cultures were interpreted without knowledge of IGRA results.

### Quality control

The JCRC laboratory is the central laboratory for the National Institutes of Health (NIH)-funded HIV Vaccine Prevention Trials Network, and the technicians have over 10 years of experience performing ELISPOT assays. Study investigators (AC and HC) received on-site training in the performance of the T-SPOT*.TB *assay from the manufacturer. These investigators conducted T-SPOT*.TB *training of JCRC technicians and supervised the T-SPOT*.TB *assays for the study.

### Statistical analysis

We performed bivariate analyses using the chi-squared test for dichotomous variables and the Mann-Whitney rank-sum test for continuous variables. We calculated sensitivity and specificity of all diagnostic tests using mycobacterial culture results as a reference standard. We compared the sensitivity and specificity of sputum AFB smear and IGRA using McNemar's test. To determine the clinical utility of IGRAs for the diagnosis of active pulmonary TB, we determined the post-test probability of TB. In this analysis, we considered the pre-test probability of TB to be equal to the TB prevalence in the study population. We then calculated likelihood ratios for positive and negative IGRA results as the proportion of patients with a given result in those with disease over the proportion of patients with the result in those without disease. Finally, we determined the post-test probability of TB by converting the pre-test probability of TB to odds, multiplying by the likelihood ratio, and converting the resulting post-test odds back to probability. We performed all analyses using STATA 10.0 (Stata Corporation, College Station, Texas), with the level of significance specified in reference to a two-tailed, type I error (p-value) less than 0.05.

## Results

### Study population

**Figure 1:**
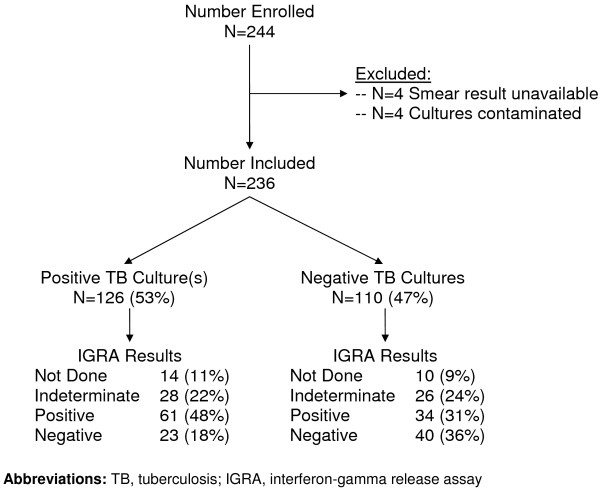
**Study population**. Of 244 patients eligible for the study, 236 (97%) were included. Pulmonary tuberculosis, defined as ≥ 1 positive culture result, was diagnosed in 126 (53%) patients.

Of 244 eligible patients enrolled, 8 (3%) were excluded from the analysis because either AFB smear results were unavailable (n = 4) or mycobacterial cultures were contaminated (n = 4) (Figure 1). Baseline characteristics (gender, age, antiretroviral use, CD4+ T-lymphocyte count were not significantly different between excluded and included patients (p > 0.2 for all comparisons).

The reference standard outcome of culture-positive TB was confirmed in 126 of 236 (53%) patients (Table 1). The diagnosis was confirmed by sputum culture in 123 patients and BAL fluid culture in 3 patients. CD4+ T-lymphocyte counts were below 200 cells/μl in greater than 75% of patients overall. However, median CD4+ T-lymphocyte counts were approximately twice as high in patients without TB compared to those with TB (75 cells/μl vs. 39 cells/μl, p = 0.01). Baseline characteristics were otherwise similar between the two groups.

**Table 1:** Demographic and clinical characteristics.

Characteristic	Overall (N = 236)	Tuberculosis (N = 126)	No Tuberculosis (N = 110)	p-value
Male, N (%)	112 (47)	61 (48)	51 (46)	0.80

Median age (IQR)	33 (27-40)	32 (27-39)	34 (27-41)	0.25

Median CD4+ T-lymphocyte count (IQR)	49 (16-160)	39 (15-107)	75 (19-215)	0.01

Anti-retroviral use, N (%)	40 (17)	17 (13)	23 (21)	0.16

In-hospital mortality, N (%)	24 (10)	13 (10)	11 (10)	1.0

Abbreviations: IQR, inter-quartile range

### IGRA results

IGRAs were not performed in 24 (10%) patients with an inadequate number of PBMCs. IGRA results were indeterminate in 54 patients (25%) either due to a low mitogen response (n = 7) or high background response (n = 47). The proportion of indeterminate test results declined significantly when comparing patients with a CD4+ T-lymphocyte count ≤ 50 cells/μl (33/109, 30%), 51-200 cells/μl (15/60, 25%), and > 200 cells/μl (6/43, 14%) (p = 0.03). Other demographic and clinical characteristics (gender, age, antiretroviral use, TB status) were also similar in patients with and without indeterminate IGRA results (p > 0.2 for all comparisons). Patients with indeterminate results had higher in-hospital mortality (17% vs. 8%, p = 0.05).

**Figure 2:**
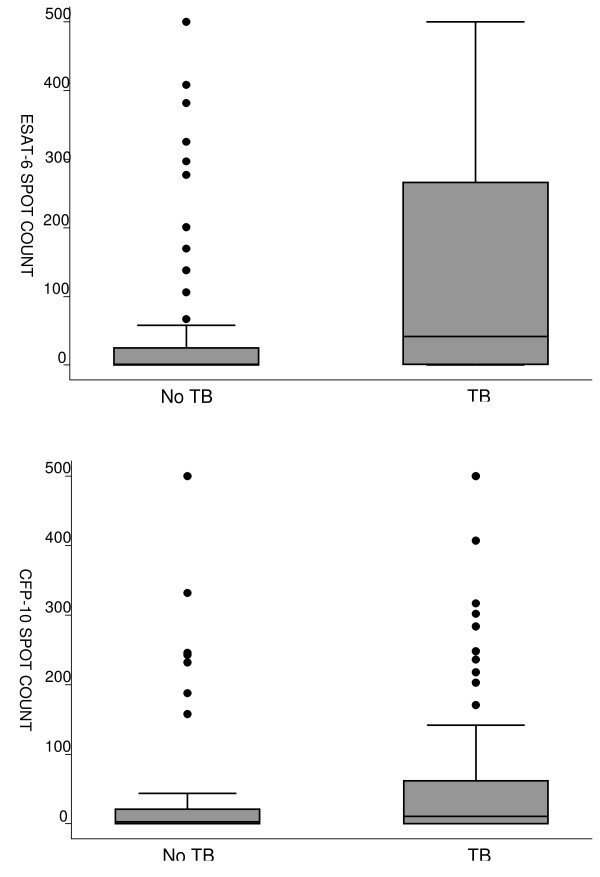
**ESAT-6 and CFP-10 spot counts in patients with and without tuberculosis**. The box plots show the distribution of spot forming cells after stimulation with ESAT-6 (top panel) and CFP-10 (bottom panel). Median spot counts were significantly higher in patients with active TB compared to those without active TB after both ESAT-6 (42 vs. 2, p < 0.001) and CFP-10 stimulation (11 vs. 3, p = 0.03).

IGRAs were positive in 95 of 158 (60%) patients with interpretable results. There was no difference in the proportion of positive test results among patients with a CD4+ T-lymphocyte count ≤ 50 cells/μl (44/76, 58%), 51-200 cells/μl (30/45, 67%), and > 200 cells/μl (21/37, 57%) (p = 0.57). Median spot counts were significantly higher in patients with active TB compared to those without active TB after both ESAT-6 (42 vs. 2, p < 0.001) and CFP-10 stimulation (11 vs. 3, p = 0.03) (Figure 2).

### Diagnostic performance

For diagnosing active pulmonary TB, IGRA sensitivity was 73% (95% CI 62-82%) and specificity 54% (42-66%) (Table 2). Sensitivity (78% vs. 67%, difference 11%, 95% CI -8% to +29%, p = 0.27) was higher among AFB smear-positive compared to smear-negative patients, but this difference was not statistically significant. Specificity was similar between the two groups. Controlling for the degree of HIV-related immunosuppression by dividing spot counts by CD4+ T-lymphocyte count [9] did not improve diagnostic accuracy compared to classifying results based on spot counts alone (for ESAT-6, CFP-10, or combined ESAT-6 and CFP-10 results; p > 0.2 for all comparisons).

**Table 2:** Sensitivity and specificity of T-SPOT*.TB*

	Overall (N = 158)	AFB Smear-Positive (N = 45)	AFB Smear-Negative (N = 113)	p-value
% Sensitivity (95% CI)	73 (62-82)	78 (62-89)	67 (52-81)	0.27

% Specificity (95% CI)	54 (42-66)	50 (7-93)	54 (42-66)	0.87

Abbreviations: AFB, acid-fast bacilli.

We next assessed the diagnostic utility of IGRAs according to AFB smear status by calculating the post-test probability of TB in patients with positive and negative IGRA results (Table 3). In this analysis, we defined pre-test probability as the TB prevalence in the study population: 94% for AFB smear-positive patients and 37% for AFB smear-negative patients. As expected, IGRA results did not meaningfully alter the probability of disease among AFB-smear positive patients. A positive IGRA result increased the probability of TB from 94% to 96% while a negative IGRA result decreased the probability of TB from 94% to 87%. More importantly, IGRA results did not contribute to a clinically meaningful change in the post-test probability of TB in AFB smear-negative patients. A positive IGRA result increased the probability of TB from 37% to 47% while a negative IGRA result decreased the probability of TB from 37% to 26%.

**Table 3:** Clinical utility of T-SPOT*.TB *in addition to AFB smear microscopy

Test Result	Likelihood Ratio	Post-test Probability of Tuberculosis
AFB Smear Positive Patients*		

T-SPOT*.TB *+	1.56	96%

T-SPOT*.TB *-	0.44	87%

AFB Smear Negative Patients^†^		

T-SPOT*.TB *+	1.48	47%

T-SPOT*.TB *-	0.6	26%

Abbreviations: AFB, acid-fast bacilli.

* Pre-test probability of tuberculosis = 94% (tuberculosis prevalence in AFB smear positive patients)

† Pre-test probability of tuberculosis = 37% (tuberculosis prevalence in AFB smear negative patients)

## Discussion

In this study, we found that an ELISPOT-based IGRA was positive in 60% of patients in a Ugandan sample of hospitalized, HIV-infected patients, indicating a high prevalence of M. TB infection. IGRA positivity was not affected by CD4+ T-lymphocyte count in our population with advanced immunosuppression. The test had moderate to high sensitivity but poor specificity in the diagnosis of active pulmonary TB. In AFB smear-negative patients, negative IGRA results did not lead to a clinically meaningful decrease in the probability of active disease. In low-income countries with high HIV prevalence, these findings suggest a role for IGRAs in LTBI screening programs but not as a rule-out test for active TB in symptomatic patients.

Numerous studies have evaluated the sensitivity of T-SPOT.TB - the only commercially available ELISPOT-based IGRA - for diagnosis of active pulmonary TB and results have been similar in immunocompetent (83-100%) and HIV-infected patients (85-93%) [7,8,10,12,16-19]. There are at least two potential explanations for the slightly lower sensitivity (73%) seen in our study. The spectrum of diseased and non-diseased patients enrolled in many studies differed from that seen in clinical practice, a factor known to inflate sensitivity estimates [20,21]. In addition, our HIV-infected study population was hospitalized and severely immunosuppressed, with more than 75% patients having a CD4+ T-lymphocyte count < 200 cells/μl. Of note, sensitivity was not increased by forming a ratio between ESAT-6 or CFP-10 spot counts and CD4+ T-lymphocyte count, an area of contention in the literature [8,9].

The specificity of T-SPOT.TB for active pulmonary TB has ranged from 45-95%, with studies in both low burden[8,10,17,18] and high burden[22] countries reporting values below 65%. The low specificity (54%) for active TB in our setting is not surprising given the known high prevalence of LTBI in Kampala. Current generation interferon-gamma release assays cannot distinguish immune responses among patients with latent infection, prior active disease, and current active disease when performed using peripheral blood specimens [2]. Further studies are needed to determine whether local immune responses can better distinguish active from latent infection[18,23-25] and to identify antigens that elicit immune responses more specific for current active disease [23].

A diagnostic test should substantially change the pre-test probability of disease and influence treatment decisions in order to have clinical utility. When evaluating TB suspects with negative AFB smears, clinicians are faced with a decision to either initiate or withhold empiric therapy. IGRAs could be useful in this situation if a negative result decreased the probability of TB below a threshold at which most clinicians would be comfortable withholding treatment. Based on the test characteristics reported in our study, a negative IGRA result would decrease the probability of TB below 10% only when the prevalence of TB among smear-negative patients is already at or below 15%. Moreover, a high proportion of indeterminate results further limited the clinical utility of IGRAs in our setting.

Our study has several potential limitations. First, the proportion of indeterminate results is higher than in previous studies despite stringent quality control procedures. Higher background levels of non-specific immune activation have been observed in Uganda compared to North American cohorts (David M. Lewinsohn, personal communication). It is also possible that non-specific immune activation was more common in our hospitalized population with advanced illness. Second, mycobacterial culture on Lowenstein-Jensen media is an imperfect reference standard. A more sensitive culture technique (such as liquid culture) may have decreased the number of false positive IGRA results and increased the number of true positive results, resulting in higher sensitivity and specificity [26]. In addition, we did not perform speciation tests to exclude the possibility of isolating non-tuberculous mycobacteria. Finally, our study population consisted of hospitalized patients with advanced HIV infection. The point estimates of sensitivity and specificity reported here may not generalize to ambulatory or predominantly HIV-uninfected populations. Nevertheless, our findings are consistent with previous studies.

## Conclusions

An ELISPOT-based IGRA detected a high prevalence of M. TB infection in a population of TB suspects with advanced HIV infection in Uganda. These assays should be considered in sub-Saharan Africa when screening HIV-infected patients for latent tuberculosis infection. However, current ELISPOT-based IGRAs are unlikely to have clinical utility when evaluating active TB suspects. Further research and innovation is needed to identify M. tuberculosis antigens and host markers that are more sensitive and specific for active tuberculosis.

## Competing interests

The authors declare that they have no competing interests.

## Authors' contributions

AC conceived of the study, participated in study design, conducted the data analysis, and drafted the manuscript. IS coordinated the immunoassays and helped draft the manuscript. JLD participated in data analysis and helped draft the manuscript. LH helped conceive of the study and participated in drafting the manuscript. WW participated in design of the study and obtained regulatory approvals. SD supervised study enrollment. SY participated in study design. AA supervised specimen collection and processing. PB collected and processed specimens. PH helped with design of the study. HC helped conceive of the study, helped coordinate the immunoassays, and helped draft the manuscript. All authors read and approved the final manuscript.

## Pre-publication history

The pre-publication history for this paper can be accessed here:

http://www.biomedcentral.com/1471-2334/10/75/prepub
